# Selenium prevents microparticle-induced endothelial inflammation in patients after cardiopulmonary resuscitation

**DOI:** 10.1186/s13054-015-0774-3

**Published:** 2015-02-26

**Authors:** Katrin Fink, Monica Moebes, Caroline Vetter, Natascha Bourgeois, Bonaventura Schmid, Christoph Bode, Thomas Helbing, Hans-Jörg Busch

**Affiliations:** Department of Emergency Medicine, University hospital of Freiburg, Sir-Hans-A.-Krebs-Str., 79106 Freiburg im Breisgau, Germany; Department of Cardiology and Angiology I, University Heart Center Freiburg, Hugstetter Str. 55, 79106 Freiburg im Breisgau, Germany

## Abstract

**Introduction:**

Microparticles are elevated in patients after successful cardiopulmonary resuscitation (CPR) and may play a role in the development of endothelial dysfunction seen in post-cardiac arrest syndrome (PCAS), a life threatening disease with high mortality. To identify mechanisms of endothelial activation and to develop novel approaches in the therapy of PCAS, the impact of selenium, a trace element with antioxidative properties, was characterized in endothelial dysfunction induced by microparticles of resuscitated patients. Additionally, course of plasma selenium levels was characterized in the first 72 hours post-CPR.

**Methods:**

Endothelial cells were exposed to microparticles isolated of the peripheral blood of resuscitated patients, and leukocyte-endothelial interaction was measured by dynamic adhesion assay. Expression of adhesion molecules was assessed by immunoblotting and flow chamber. Blood samples were drawn 24, 48 and 72 hours after CPR for determination of plasma selenium levels in 77 resuscitated patients; these were compared to 50 healthy subjects and 50 patients with stable cardiac disease and correlated with severity of illness and outcome.

**Results:**

Microparticles of resuscitated patients enhance monocyte-endothelial interaction by up-regulation of ICAM-1 and VCAM-1. Selenium administration diminished ICAM-1 and VCAM-1-mediated monocyte adhesion induced by microparticles of resuscitated patients, suggesting that selenium has anti-inflammatory effects after CPR. Lowered selenium plasma levels were observed in resuscitated patients compared to controls and selenium levels immediately and 24 hours after CPR, inversely correlated with clinical course and outcome after resuscitation.

**Conclusions:**

Endothelial dysfunction is a pivotal feature of PCAS and is partly driven by microparticles of resuscitated patients. Administration of selenium exerted anti-inflammatory effects and prevented microparticle-mediated endothelial dysfunction. Decline of selenium was observed in plasma of patients after CPR and is a novel predictive marker of ICU mortality, suggesting selenium consumption promotes inflammation in PCAS.

**Electronic supplementary material:**

The online version of this article (doi:10.1186/s13054-015-0774-3) contains supplementary material, which is available to authorized users.

## Introduction

The worldwide estimated annual incidence of sudden cardiac death is in the range of four to five million cases per year. Despite improved treatment, median survival rate to hospital discharge remains low, from 8 to 18% [1,2]. The poor prognosis of patients after cardiopulmonary resuscitation (CPR) is caused by a post-cardiac arrest syndrome (PCAS), a combination of brain injury, circulatory dysfunction and systemic ischemia/reperfusion injury [3,4]. Important steps in the pathogenesis of PCAS are the release of reactive oxygen species (ROS) and endothelial-leukocyte interaction [5], resulting in a systemic inflammatory response [5,6], endothelial activation and injury [7-9] and coagulation abnormalities [7,10].

Microparticles (MPs) are small vesicles (0.1 to 1.5 μM) [11] that originate from vesiculation of the cell membrane of most eukaryotic cells undergoing activation or apoptosis [12], thereby expressing antigens characteristic of their cell of origin [13]. MPs are considered to act as diffusible messengers [14] to transport bioactive agents and seem to have gene-regulatory function [15]. MPs may initiate and mediate coagulation [16], inflammation and cell-cell interactions [12]. They are able to activate platelets, leukocytes and endothelial cells [17,18] and are known to participate in a variety of intercellular adhesion processes [17] and to induce endothelial dysfunction [19,20]. Recently our group has shown that resuscitated patients show substantially increased levels of different microparticles [8,21] and that those microparticles may contribute to the pathogenesis of PCAS by induction of endothelial apoptosis [21].

Selenium is a nutritional trace element essential for various aspects of human health, exerting its effects mainly through its incorporation into selenoproteins. Furthermore, selenium has been reported to modulate cytokine-induced expression of adhesion molecules on endothelial cells [22,23]. Accordingly, selenium deficiency enhances neutrophil adhesion to endothelial cells [24]. Numerous studies report protective effects of selenium against ischemia/reperfusion injury [25-27] by reduction of ROS, thus decreasing the inflammatory and endothelial reactions [27].

Critically ill patients who suffer from severe oxidative stress exhibit low selenium plasma levels [28,29]. Supporting this notion, selenium substitution has been shown to reduce mortality in patients with severe sepsis or septic shock [30,31].

The objective of the present study was to determine the impact of microparticles of patients after successful cardiopulmonary resuscitation on endothelial cells *ex vivo* and to test the hypothesis that selenium may exert endothelial protective effects. Furthermore we aimed to characterize the time course of plasma selenium concentrations in resuscitated patients. We hypothesized that decrease in plasma selenium levels may contribute to development of post-cardiac arrest syndrome, organ failure and adverse outcome.

## Material and methods

### Isolation of circulating MPs from resuscitated patients and healthy volunteers

Microparticles were isolated out of 25 mL of whole blood collected in citrated tubes from resuscitated patients 24 hours after return of spontaneous circulation (ROSC), or healthy subjects, respectively. Samples were drawn slowly, handled carefully and were processed immediately after sampling. In the case of venipuncture, we used a 21-gauge butterfly needle and discarded the first 7.5 mL. If available, blood was drawn from an arterial or central venous catheter.

MP isolation was performed according to the protocol of Boulanger *et al*. [32]. In brief, platelet-free plasma was obtained by centrifugation at 11,000 g for two minutes and was subjected to further centrifugation at 13,000 g for 45 minutes. After resuspension of the MP pellets 100 μL of RPMI medium (containing Hepes and L-glutamine, from Lonza, Verviers, Belgium) was added and samples were frozen at −80°C until further analysis. Supernatants, containing the corresponding microparticle-free dilution of subjects, were also stored at −80°C and served as negative controls. MP protein content was assessed by Bio-Rad Protein Assay (Bio-Rad Laboratories GmbH, Munich, Germany) and adjusted to the same values for each subgroup in the experiments. Protein content of pellets and microparticle-free supernatant did not differ in resuscitated patients (MP: 15.54 ± 2.581 versus supernatant: 17.08 ± 2.202 μg/μL, *P* = 0.6540 (not significant)) and healthy controls, suggesting a comparability of each pellet with its corresponding supernatant.

### Cell culture of human umbilical vein endothelial cells (HUVECs)

HUVECs were obtained from Promocell^TM^ (Heidelberg, Germany). The cells were cultured in endothelial cell growth medium advanced (Provitro, Berlin, Germany), containing 10% fetal calf serum (FCS), Heparin (22.50 μg), human recombinant epidermal growth factor (5 ng), human recombinant fibroblast growth factor (10 ng), human recombinant vascular endothelial growth factor (0.5 ng), human recombinant insulin-like growth factor-1 (20 ng), ascorbic acid (1 μg), hydrocortisone (0.20 μg), gentamicin (50 μg), L-glutamine (2 mmol), and cell culture plastic was from Nunc (Rolkilde, Denmark). Cultures were kept at 37°C in a 5% CO_2_ humidified atmosphere.

### Monocytes

After approval from the ethics committee of our institution (EK-Freiburg 10015/12), monocytes were isolated from buffy coat leukocytes or citrated human blood from healthy volunteers. Monocytes were isolated by Ficoll (Biocoll Separating Solution, Biochrom, Berlin, Germany) gradient centrifugation and plastic adhesion. Cells were mixed 1:1 (in case of blood separation) or 1:5 (in case of buffy coat separation) with PBS (Lonza, Veriers, Belgium). After centrifugation at 800 × g for 20 minutes at room temperature the intermediate layer of cells was removed and washed with PBS. Cells were maintained in RPMI medium (Invitrogen, Paisley, UK) with 10% FCS, 1% non-essential amino acids, 2 mmol L-glutamine, 100 U/mL penicillin and 100 μg/ mL streptomycin.

### Antibodies and reagents

The following fluorescence-labelled antibodies were used: anti-human ICAM-1 (CD54) antibody (R&D Systems, Minneapolis, MN, USA); anti-human VCAM-1 (CD106) antibody (R&D System); anti-mouse IgG1 Cy2 secondary antibody (for ICAM-1) (Acris Antibodies, Herford, Germany) and anti-mouse IgG Cy3 secondary antibody (for VCAM-1) (Jackson ImmunoResearch Laboratories, Baltimore Pike, MD, USA). Antibodies used for western blot were: anti-human ICAM-1 (CD54) antibody (Cell Signaling Technologies, Danvers, MA, USA); anti-human VCAM-1 (CD106) antibody (Santa Cruz Biotechnology, Dallas, Texas, USA); secondary anti-mouse IgG-HRP antibody (R&D Systems) and secondary anti-rabbit IgG-HRP antibody (R&D Systems, Minneapolis USA). Vybrant^TM^ carboxyfluorescein-diacetate (CFDA) (Invitrogen, Darmstadt, Germany) was used for staining of monocytes in flow chamber experiments.

### Dynamic adhesion assay and endothelial blocking by selenium

HUVECs were grown in 35-mm dishes (Costar, Bethesda, MD, USA) to 100% confluence. The Glycotech (Gaithersburg, MD, USA) flow chamber was assembled with the dish as the bottom of the resulting parallel flow chamber. The chamber and tubes were filled with PBS prior to the experiment. Subsequently, isolated human monocytes were applied with a syringe and shear stress was induced with a syringe pump (Harvard apparatus PHD2000, Holliston, MA, USA) with a flow rate of 0.25 dyne/cm^2^ (venous flow) for a total of 10 minutes, and then with 15 dyne/cm^2^ (arterial flow) for 1 minute. Monocytes were allowed to adhere to the endothelial cell layer after pre-treatment of HUVECs for 6 h either with microparticles of resuscitated or healthy subjects, or with the cell free supernatant of resuscitated patients, respectively. In case of selenium treatment HUVECs were pre-incubated with 1.5 μM selenium for 24 h additionally.

Adherent cells were quantified under the microscope and monocytes were visualized by CFDA staining (500 μL CFDA per 70*10^6^ monocytes, incubated for 15 minutes at 37°C in a water bath). Data from at least four different experiments were analyzed.

### Immunocytochemistry

HUVECs were seeded on glass coverslips. Cells were incubated for 6 h with microparticles from resuscitated or healthy subjects, or with the cell free supernatant of resuscitated patients, respectively. Cells were fixed in ice-cold methanol/acetone at −20°C for 10 minutes and were blocked with 10% donkey serum for 30 minutes at room temperature. Subsequently HUVECs were incubated with an anti-human ICAM-1, or VCAM-1 antibody (both from R&D Systems) and an anti-mouse IgG1 Cy2 secondary antibody for ICAM (Acris Antibodies GmbH), or an anti-mouse IgG Cy3 secondary antibody for VCAM (Jackson ImmunoResearch Laboratories Inc.), respectively. In the case of selenium treatment HUVECs were additionally pre-incubated with 1.5 μM selenium for 24 h.

### Western blot

HUVECs were stimulated for 6 h with microparticles from resuscitated or healthy subjects, or with the cell free supernatant of resuscitated patients, respectively, in endothelial cell growth medium (Provitro) with 10% fetal bovine serum (FBS). Cell lysates were resolved on a reducing polyacrylamide gel, plotted onto a nitrocellulose membrane (Amersham GE Healthcare, Pittsburgh, PA, USA) and blocked with 3% non-fat milk in PBS/Tris with 0.1% Tween 20 for 2 h at room temperature (20 to 22°C). The membrane was then incubated with primary antibody overnight at 4°C. After 1 h of incubation with the secondary antibody, proteins were visualized using ECL reagent (Amersham GE Healthcare). Densitometric analysis of western blots was performed using Quantity One 1-D Analysis Software Version 4.4 (Bio-Rad Laboratories GmbH, Munich, Germany). All western blots were repeated at least three times and quantified data are shown.

### Measurement of plasma selenium levels

For measurements of selenium plasma levels 2 to 3 mL of whole blood were collected in EDTA tubes every 24 h from day one to three after ROSC (day 1, 2, 3). Samples were stored at −20°C. Measurements of selenium concentrations were performed blinded by staff in the laboratories of Biosyn (Fellbach, Germany) by electrothermal atomic absorption spectrometry using a spectrometer equipped with a Zeeman effect background correction (5100 PC, Perkin-Elmer, Paris, France).

Samples for measurement of routine indicators of organ dysfunction/failure (for example, creatinine, diuresis, bilirubin, albumin, platelet count, blood pressure, catecholamine doses, or arterial pressure of oxygen/inspired oxygen fraction (paO_2_/FiO_2_) were simultaneously collected. Measurements were performed in the laboratories of the University hospital of Freiburg using automated measures.

### Patient recruitment

After the institutional approval of the Ethics Committee of Albert Ludwigs University (EK-Freiburg 328/09) we took blood samples for microparticle isolation from a total of 77 patients who underwent CPR after cardiac arrest from any cause. All patients were treated according to the international guidelines given by International Liaison Committee on Resuscitation in 2008 [4]. Results were compared with 50 healthy subjects without any cardiovascular risk who were taking no medication.

Because lowered selenium plasma levels are described in patients with coronary artery disease (CAD) [33,34] we chose a second cardiac control group to exclude possible effects on selenium levels caused by underlying cardiac disease. We therefore included 50 patients with stable cardiac disease who were admitted to a cardiological ward for any reason for measurements of selenium plasma levels.

### Inclusion and exclusion criteria

Inclusion criteria were CPR following cardiac arrest due to any cause, occurring in or out of hospital, and with a duration of at least five minutes. Resuscitation was performed according to the European Resuscitation Council Guidelines for Resuscitation [4]. Patients with malignant diseases were excluded from the study, because elevation of MPs has been reported to be increased in various malignancies [35]. Similarly, patients younger than 18 years, pregnant patients, patients with sepsis or severe inflammation or trauma patients were excluded, because alterations in plasma selenium levels have already been described in these patients [36]. Informed consent was obtained post hoc from patients surviving with good neurological outcome or from their relatives in the case of non-surviving patients. Informed consent was given by all patients in the control group. All patients received micronutrient supplementation containing 0.032 mg per day of selenium, no later than 24 h after admittance to the ICU. Twenty-three patients had to be excluded retrospectively because of selenium administration (1000 μg daily) in the first 72 h after CPR.

### Data collection

Baseline characteristics were recorded including age, gender, concomitant diseases and cardiovascular risk profile. In resuscitated patients data on cardiac arrest and cardiopulmonary resuscitation were registered and severity of disease was evaluated by sequential organ failure assessment (SOFA) [37]. The SOFA score was calculated daily and the maximum SOFA score was defined as the highest score reached during the first 3 days of the ICU stay.

### Statistical analysis

Continuous variables are expressed as mean ± standard error of the mean (SEM). Because data for CPR, control patients and healthy subjects were normally distributed, they were compared by Student’s *t*-test. Correlations between selected variables were estimated by Spearman-rho test and the corresponding *R*^2^ was calculated. The predictive value of plasma selenium concentrations on ICU outcome was calculated using a receiver operating characteristic (ROC) curve and the area under the curve (AUC) was computed and analyses were performed with SPSS version 19.0 (SPSS Inc., Chicago, IL, USA). In flow chamber experiments a correction for multiple testing was made by two-way repeated-measures analysis of variance (ANOVA) with post-test Bonferroni correction, including an all-pair-wise comparison. Basic data and outcomes were compared by chi-quadrate test, Fisher’s exact test, and Student’s *t*-test, respectively. Statistical significance was defined as a two-tailed *P*-value <0.05.

## Results

### Microparticles of resuscitated patients enhance monocyte-endothelial interaction under flow conditions

To assess the impact of MPs from resuscitated patients on endothelial inflammation, cultured HUVECs were exposed to MPs and monocyte-endothelial adhesion was quantified under physiological flow conditions. Supporting the notion that MPs mediate inflammatory effects, endothelial cell exposed to MPs from resuscitated patients increased monocyte adhesion to endothelial cells compared to exposure to the corresponding microparticle-free supernatant of each MP sample from CPR patients in a dynamic adhesion assay (after 5 minutes: MP: 0.38 ± 0.05 versus supernatant: 0.24 ± 0.04 adherent monocytes per HUVEC, *P* = 0.02; after 10 minutes: MP: 0.64 ± 0.09 versus supernatant: 0.39 ± 0.05 adherent monocytes per HUVEC, *P* = 0.02 and after 1 minute of arterial flow: MP: 0.47 ± 0.04 versus supernatant: 0.28 ± 0.06, *P* = 0.01).

**Figure 1:**
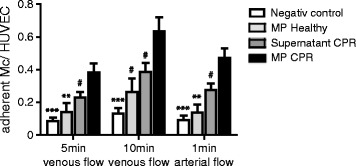
**Microparticles from resuscitated patients enhance monocyte-endothelial interaction under flow conditions.** Stimulation of human umbilical vein endothelial cells (HUVECs) with microparticles from resuscitated patients (black bars) leads to enhanced monocyte-endothelial interaction under venous flow (left and middle), and firm adherence under arterial flow (right) conditions in the dynamic adhesion assay, compared to microparticle-free supernatant from resuscitated patients (dark gray bars) and stimulation with microparticles from healthy subjects (light gray bars): ***P <0.0001; **P <0.001; ^#^ *P* <0.05; ns = not statistically significant). CPR, cardiopulmonary resuscitation; ICAM-1, intracellular adhesion molecule-1; VCAM-1, vascular cell adhesion molecule-1.

Additionally MPs from resuscitated patients augmented monocyte adhesion 2.4-fold when compared with the effect of MPs from healthy subjects (after 5 minutes: healthy: 0.14 ± 0.05 adherent monocytes per HUVEC, *P* = 0.009; after 10 minutes: healthy: 0.26 ± 0.09, *P* = 0.01 and after 1 minute of arterial flow: healthy: 0.14 ± 0.05, *P* = 0.0009) (Figure 1).

Taken together these data suggest that MPs from resuscitated patients enhance monocyte-endothelial interaction under flow conditions.

### Microparticles of resuscitated patients increase expression of endothelial adhesion molecules

**Figure 2:**
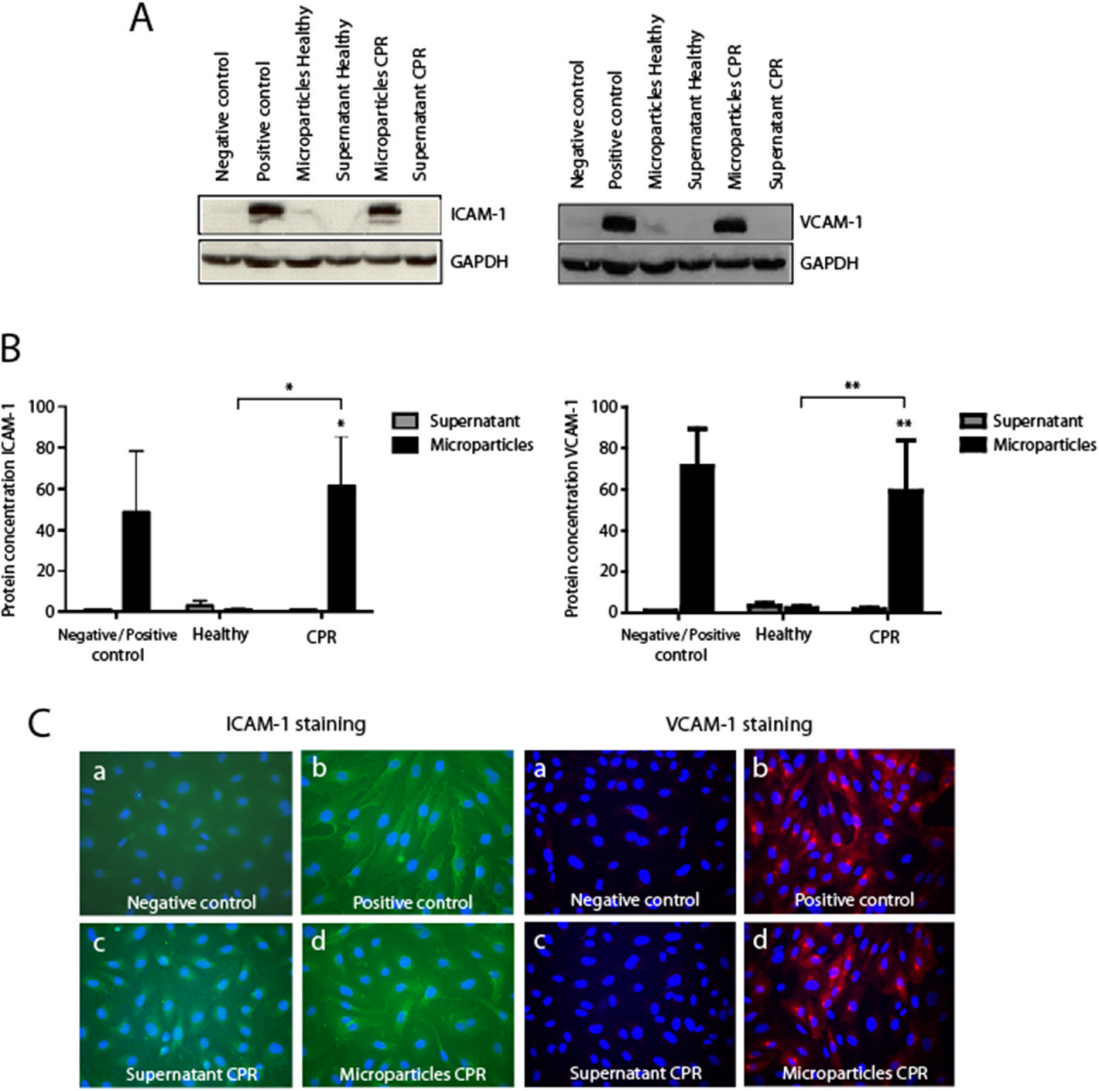
**Microparticles from resuscitated patients increase expression of intracellular adhesion molecule-1 (ICAM-1) vascular cell adhesion molecule-1 (VCAM-1) on endothelial cells.** **(A,** **B)** Increased ICAM-1 (left panels) and VCAM-1 (right panels) protein expression after endothelial stimulation with microparticles (black bars on the right in each graph) compared with microparticle-free supernatant from resuscitated patients (gray bars; right), shown by western blot analysis and compared with stimulation with microparticles (black bars in the middle of each graph) and microparticle-free supernatant (gray bars; middle) from healthy subjects. Positive and negative controls are shown on the left in each panel. **(C)** Increased ICAM-1 (panels on the left; green staining by Cy2) and VCAM-1 (panels on the right; red staining by Cy3) protein expression after human umbilical vein endothelial cell (HUVEC) stimulation with microparticles from resuscitated patients (lower right panels in each illustration), shown by immunocytochemistry, compared with microparticle-free supernatant from resuscitated patients (lower left panels) and negative control (upper left panels): 4,6-Diamidino-2-phenylindole (DAPI) was used for staining of nuclei. (**P <0.001; *P <0.01). CPR, cardiopulmonary resuscitation.

To evaluate the molecular mechanisms leading to the increase in monocyte adhesion to the endothelium following exposure to MPs of resuscitated patients, we investigated expression of ICAM-1 and VCAM-1 after stimulation of HUVECs with MPs. Both immunocytochemistry and western blot experiments revealed that endothelial cell exposure to MPs of CPR patients increased the levels of ICAM-1 expression of endothelial cells (Figure 2), compared to MP-free supernatant and MPs from healthy subjects.

Consistent with regulation of ICAM-1 endothelial cell exposure to MPs from CPR patients increased the levels of VCAM-1 expression of endothelial cells, shown by immunocytochemistry and western blot experiments (Figure 2). As expected, MP-free supernatant, as highly diluted plasma solution did not significantly enhance ICAM-1 and VCAM-1 expression at the endothelium side. These findings suggest that adhesion molecules ICAM-1 and VCAM-1 participate in MP-induced endothelial monocyte interaction.

### Selenium reduces monocyte adhesion to the endothelium under flow conditions

**Figure 3:**
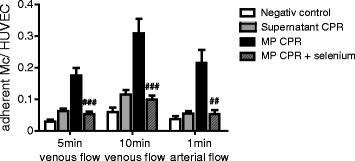
**Selenium administration reduces monocyte adhesion to the endothelium under flow conditions.** Addition of selenium on human umbilical vein endothelial cells (HUVECs) diminished microparticles-induced monocyte-endothelial interaction (crosshatched bars) after stimulation with microparticles from resuscitated patients under venous flow (left and middle), as well as firm adherence under arterial flow (right) conditions in dynamic adhesion assay, compared with unblocked stimulation with microparticles from resuscitated patients (black bars) and to microparticle-free supernatant from resuscitated patients (dark gray bars). (**P <0.001; *P <0.01).

The increased interaction of monocytes to the endothelium after stimulation with MPs from CPR patients in the dynamic adhesion assay can be completely blocked by pre-treatment of endothelial cells with selenium under venous flow (after 5 minutes, no selenium, 0.18 ± 0.02 versus selenium, 0.05 ± 0.01 adherent monocytes per HUVEC, *P* = 0.0004; after 10 minutes, no selenium, 0.31 ± 0.05 versus selenium, 0.1 ± 0.01, *P* = 0.0002) and after 1 minute of arterial flow (no selenium, 0.22 ± 0.04 versus selenium, 0.05 ± 0.01, *P* = 0.001) (Figure 3).

This result demonstrates that selenium reduces monocyte adhesion to the endothelium under physiological flow conditions.

### Selenium administration reduces expression of endothelial adhesion molecules after stimulation with MP of resuscitated patients

**Figure 4:**
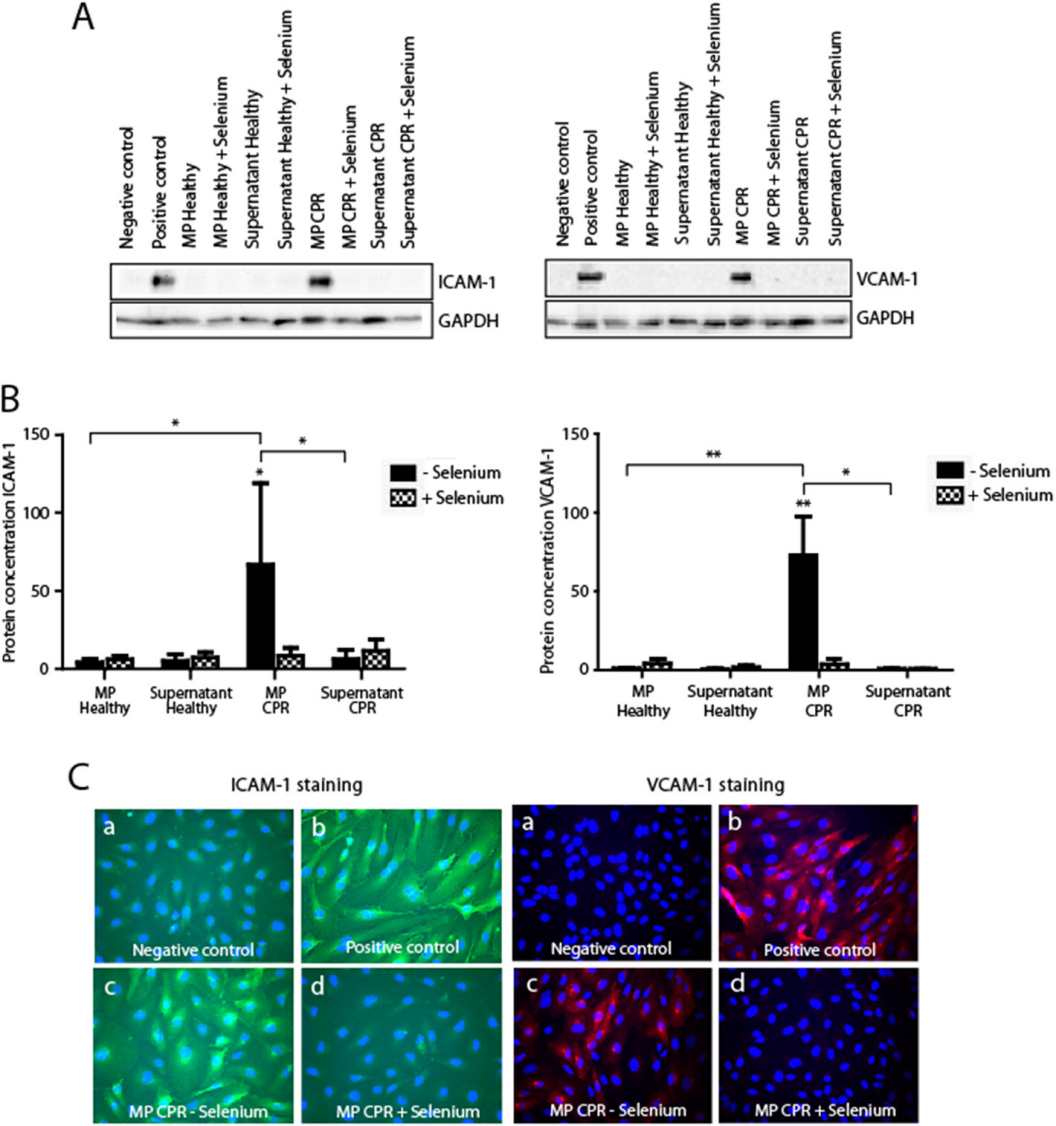
**Selenium administration reduces expression of intracellular adhesion molecule-1 (ICAM-1) and vascular cell adhesion molecule-1 (VCAM-1) after stimulation with microparticles.** **(A, B)** Addition of selenium on human umbilical vein endothelial cells (HUVECs) diminished microparticle-induced ICAM-1 (left panels) and VCAM-1 (right panels) protein expression (checkered bars in each panel), shown by western blot analysis, compared with unblocked stimulation with microparticles or supernatant (black bars) from resuscitated patients (on the right in each panel) healthy controls (on the left), respectively. **(C)** Addition of selenium on HUVECs diminished microparticle-induced ICAM-1 (left panels; green staining by Cy2) and VCAM-1 (right panels; red staining by Cy3) protein expression after HUVEC stimulation with microparticles from resuscitated patients (lower right panels in each illustration), shown by immunocytochemistry, compared with unblocked stimulation with microparticles from resuscitated patients (lower left panels): 4,6-Diamidino-2-phenylindole (DAPI) was used for staining of nuclei. (**P <0.001; *P <0.01).

To evaluate if reduced monocyte adhesion is a consequence of reduced expression of adhesion molecules, we tested ICAM-1- and VCAM-1- expression of MP-stimulated endothelial cells after selenium administration. The augmented expression of the endothelial adhesion molecules ICAM-1 and VCAM-1 after stimulation of endothelial cells with MPs from CPR patients can be completely blocked after selenium pre-treatment, shown by immunocytochemistry and western blot experiments (Figure 4).

Taken together selenium administration reduces expression of endothelial adhesion molecules of endothelial cells stimulated with MP of resuscitated patients.

### Selenium plasma levels are diminished in resuscitated patients

To assess the role of selenium in post cardiac arrest syndrome, levels of selenium were quantified in patients (n = 77) after successful cardiopulmonary resuscitation. As shown in Table 1 baseline characteristics of resuscitated patients are comparable to those published in other studies in view of age, duration and cause of CPR, severity of illness and outcome parameters.

**Table 1:** **Baseline characteristics of resuscitated and control patients**

		**CPR (n = 77)**		**Cardiac control (n = 50)**		***P- value***	**Healthy (n = 50)**		***P-value***
		***Number***	***%***	***Number***	***%***	***(versus CPR)***	***Number***	***%***	***(versus CPR)***
**Age (years)**		64.7 ± 1.9		69.9 ± 1.7		**<0.05**	30.4 ± 1.4		**<0.001**
**Gender**	Male	59	77.6	33	66.7	0.22/ **ns**	24	48.0	**<0.01**
	Female	18	23.4	17	33.3		26	52.0	
**Cause of cardiac arrest/hospital admission**	Cardiac	58	75.3	50	100.0	**<0.005**			
	Non cardiac	15	19.5	0	0.0				
	Unknown	4	5.2	0	0.0				
**Medical history**	CAD	52	67.5	41	82.0	0.10/ **ns**	0	0.0	**<0.001**
	Coronary angiography	58	75.3	35	70.0	0.54/ **ns**			
	PCI	39	50.6	22	44.0	0.47/ **ns**			
**Duration of CPR (minutes)**		24.5 ± 4.9							
**Estimated duration of asphyxia (minutes)**		3.3 ± 0.5							
**Initial rhythm**	VT/ VF	49	63.6						
	Asystole/ PEA	28	36.4						
**Outcome**	Survival to discharge	41	53.2	48	96	**<0.001**			
**Length of ICU stay**		7.6 ± 0.7							
**GOS at discharge**		4.2 ± 0.2		5.5 ± 0.0		**<0.001**			
**SOFA**	within 24 h after ROSC	11.1 ± 0.3							
	within 48 h after ROSC	10.9 ± 0.4							
	within 72 h after ROSC	10.5 ± 0.4							

Table 1 shows the clinical characteristics of all resuscitated patients (n = 77), cardiac control patients (n = 50) and healthy controls (n = 50) that were included into measurement of plasma selenium levels. Cause of cardiac arrest was defined as any underlying cardiac disease leading to cardiac arrest, or hospital admission, respectively (for example, coronary artery disease (chronic or acute), heart failure, rhythmic disturbances). CPR, cardiopulmonary resuscitation; CAD, coronary artery disease; PCI, percutaneous coronary intervention; VT, ventricular tachycardia; VF, ventricular fibrillation; PEA, pulseless electrical activity; GOS, Glasgow outcome scale; SOFA, sequential organ failure assessment; ROSC, return of spontaneous circulation.

**Figure 5:**
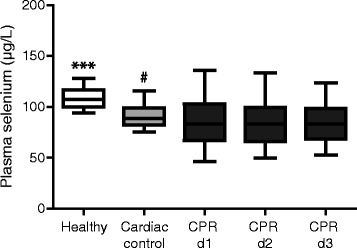
**Lowered selenium plasma levels in resuscitated patients.** Selenium plasma levels (measured by electro thermal atomic absorption spectrometry) of patients after cardiopulmonary resuscitation (CPR) are reduced in the first 72 hours after CPR (black bars; 24 hours: CPR day 1, 48 hours: CPR day 2, 72 hours: CPR day 3), compared with patients with stable cardiac disease (gray bar; coronary artery disease (CAD)) and healthy subjects (white bar; Healthy). (***P <0.0001; ^##^ *P* <0.005).

All measurements were also performed in 50 healthy subjects without a cardiological history and a mean age of 30.4 ± 1.4 years. Selenium plasma levels were also compared to a cardiac control group consisting of 50 patients that were comparable in baseline characteristics. Resuscitated patients had the lowest plasma selenium concentrations at study inclusion compared to healthy subjects (86.0 ± 2.9 versus 109.1 ± 1.3 μg/L; *P* <0.0001 and compared to the cardiac controls (versus 94.2 ± 2.2 μg/L; *P* = 0.04). Plasma selenium concentrations persisted low on the second (84.8 ± 2.9 μg/L) and third day (82.1 ± 3.1 μg/L) after ROSC compared to cardiac control patients (day 2: *P* = 0.02; day 3: *P* = 0.047) or healthy subjects (day 2: *P* <0.0001; day 3: *P* <0.0001) (Figure 5).

### Decline of plasma selenium in resuscitated patients is associated with severity of disease and poor outcome

**Figure 6:**
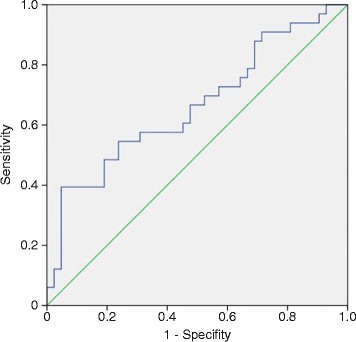
**Plasma selenium levels on day 1 are a predictive marker of ICU mortality.** Plasma selenium concentrations of resuscitated patients measured within the first 24 hours after cardiopulmonary resuscitation had predictive power in identifying ICU mortality (computing the area under the curve (AUC) = 0.665 of a receiver operating characteristic (ROC).

Resuscitated patients who survived the ICU stay (survivors) had significantly higher plasma selenium levels at admission (98.1 ± 4.5 versus 75.6 ± 3.4 μg/L; *P* = 0.0007) and on the third day after CPR (89.9 ± 4.9 versus 74.1 ± 3.4 μg/L; *P* = 0.048)., Accordingly ICU non-survivors had significantly lower plasma selenium levels compared to both control groups at all time points (versus cardiac controls: day 1 *P* = 0.00047; day 2 *P* = 0.003; day 3 *P* = 0.0006; and healthy days 1 to 3: *P* <0.0001) (data not shown). Plasma selenium concentrations of resuscitated patients measured within the first hours after CPR (day 1) had predictive power in identifying ICU mortality computing the area under the curve (AUC) of a receiver operating characteristic (ROC) (AUC = 0.665, *P* <0.05) (Figure 6).

Furthermore, the minimum plasma selenium concentration was inversely correlated to the maximal degree of organ dysfunction/failure during the ICU stay as assessed by the maximum SOFA score (*R*^2^ = 0.27, *P* <0.05) (data not shown). Taken together, reduced selenium plasma levels in resuscitated patients in the post-resuscitation period are associated with severity of disease and poor outcome.

## Discussion

In the present study we demonstrate that microparticles of patients after successful cardiopulmonary resuscitation cause enhanced monocyte adhesion to the endothelium that is partly mediated by enhanced expression of the endothelial adhesion molecules ICAM-1 and VCAM-1. Both effects can be blocked by selenium administration, suggesting a potential therapeutic approach for selenium in post-cardiac arrest syndrome. Furthermore selenium plasma levels were lowered in resuscitated patients, particularly in ICU non-survivors and selenium levels have predictive value in identifying ICU mortality.

In PCAS whole body ischemia/reperfusion leads to enhanced endothelial inflammation reflected by endothelial-leukocyte interactions [7,6], resulting in increased microvascular permeability and, hence, loss of endothelial integrity [8,9,38]. Brodsky *et al*. showed that circulating MPs directly affect the endothelium [20] and MPs isolated from patients with septic shock, or acute myocardial infarction cause endothelial dysfunction or apoptosis [19,39]. Our group recently described elevated endothelial-derived MPs in patients after CPR [8,21] and that these MPs may also enhance endothelial apoptosis [21]. Therefore, in the present study, we aimed to investigate the influence of MPs on the endothelium and monocyte-endothelial interaction *ex vivo.* Hereby we identify MPs of resuscitated patients to enhance monocyte adhesion to endothelial cells under physiologic flow conditions. This bond seems to be a foremost tight adherence, as most of the monocytes remained bound even under arterial flow conditions.

Binding is realized by enhanced ICAM-1 and VCAM-1 expression on the endothelium after exposure to MPs of CPR patients, proven by western blot and immunocytochemistry results. Barry *et al*. had already reported in 1998 that platelet MPs are able to increase monocyte adherence to endothelial cells by upregulation of adhesion molecules on both cell types [40]. Recently, the TIDE study presented evidence for different patterns of acute coronary occlusion with increased intracoronary concentrations of endothelial-derived MPs in patients suffering from cardiac arrest [41]. Unfortunately in the present study, we did not assess the MP subtypes, therefore a distinction of the action of specific subtypes of MPs generated during and after CPR is not possible.

Mechanistically, increased expression of adhesion molecules in response to MPs may be due to internalization of MPs by endothelial cells, thereby inducing expression of cell adhesion molecules and activation of the nuclear factor-κB [42]. However, isolated MPs from human atherosclerotic plaques have also been shown to transfer ICAM-1 directly to endothelial cell membranes [43]. Accordingly, we could not detect enhanced mRNA expression of endothelial adhesion molecules after MP stimulation (data not shown)], which is in line with the literature available [43]. Nevertheless, the precise mechanism of increased ICAM-1 and VCAM-1 expression in endothelial cells exposed to MP of resuscitated patients was not addressed in the present study. To exclude effects of any compounds occurring in the dilution of the MP pellets, we chose supernatant of pelleted MPs as an internal control.

As selenium has been shown to inhibit cytokine-induced expression of endothelial adhesion molecules [22], thus reducing leukocyte adhesion to the endothelium [44], we focused on selenium as a potential therapeutic approach in this setting. Indeed selenium administration significantly reduced monocyte adhesion to HUVECs stimulated by MPs from resuscitated patients, thereby protecting endothelial cells from MP-related activation. At the cellular level we demonstrated that selenium administration may inhibit the upregulation of endothelial adhesion molecules ICAM-1 and VCAM-1 caused by MPs of resuscitated patients. Other possible mechanisms of reduced leukocyte-endothelial interaction after selenium administration, such as downregulation of monocyte adhesion molecules were not addressed in the present study.

As decreased plasma selenium concentrations occur in severely ill ICU patients [28,29], we were interested in selenium plasma levels of patients after successful CPR. Actually, resuscitated patients exhibited significantly lower plasma selenium concentrations compared to healthy subjects and to control patients presenting with stable cardiac disease. Plasma levels were lowest at study inclusion but remained low up to 72 hours after ROSC. According to the literature decreased plasma selenium concentrations are found in patients with systemic inflammation [29], including surgical [36] and trauma patients [45]. Interestingly, patients undergoing cardiac surgery, most closely resembling whole body ischemia/reperfusion occurring after CPR, also exhibit lowered selenium plasma levels [29,46]. Additionally, low selenium blood levels in this study were an independent predictor for the postoperative development of multiorgan failure [46] and reduced plasma selenium concentrations were associated with increased ICU mortality in critically ill patients [29,36]. Our study revealed comparable results in resuscitated patients, as ICU non-survivors exhibited significantly lower plasma selenium levels compared to ICU survivors at all time points. Moreover, plasma selenium concentrations of resuscitated patients at admission to the ICU were predictive in identifying ICU mortality after CPR and were related to organ dysfunction/failure assessed by the SOFA score. This is in line with other publications showing a comparable inverse correlation of selenium levels to acute physiology and chronic health evaluation (APACHE) II score, simplified acute physiology score (SAPS) II and SOFA scores in critically ill patients [36].

Mechanistically, antioxidant properties of selenium incorporated into selenoproteins, such as glutathione peroxidase (GPx) [47], are exhibited by mitigating tissue damage caused by ROS [48,49]. It is well established GPx activity parallels serum selenium levels [50,51] and selenium deficiency is associated with subnormal levels of selenoproteins [52]. Accordingly, randomized controlled trials and recent meta-analyses showed that high-dose selenium treatment is effective in reducing mortality rates in critically ill patients [53], as well as patients with severe sepsis or septic shock [30,54,55]. Interestingly, no selenium-specific side effects were observed in several studies, even in high-dose selenium treatment [30,56]. Regarding the present data, one might speculate that selenium supplementation might improve survival and/or clinical course of patients after successful CPR, but this has to be addressed in randomized controlled trials.

A certain limitation of the present study might be the comparison of critically ill patients after CPR with healthy subjects. As there is an association between low selenium status and coronary artery disease [33,34], we aimed to exclude possible effects on selenium levels caused by CAD in the resuscitation group by choosing a second control group of patients with stable cardiac disease, presenting mostly CAD. It must however be mentioned that resuscitated patients were not matched with the cardiac control group, which limits the strength of the evidence.

## Conclusion

The present study provides evidence of the pathophysiological relevance of microparticles in post-cardiac arrest syndrome, as they strongly alter endothelial and vascular function and may translate ischemia/reperfusion injury into a systemic process. Selenium plasma levels in resuscitated patients are predictive of patients’ clinical course and outcome after cardiopulmonary resuscitation. For the first time we identify selenium administration as a possible therapeutic approach by mitigating endothelial activation in post-cardiac arrest syndrome.

## Key messages

- Microparticles isolated from patients after cardiopulmonary resuscitation enhance monocyte adhesion to endothelial cells *ex vivo* by upregulation of ICAM-1 and VCAM-1 on endothelial cells
- Selenium administration moderated microparticle-mediated monocyte-endothelial interaction and upregulation of ICAM-1 and VCAM-1 on endothelial cells, thereby exhibiting anti-inflammatory effects
- Patients after successful cardiopulmonary resuscitation exhibit lowered selenium plasma levels compared to healthy subjects and patients with cardiac disease
- Plasma selenium levels of resuscitated patients inversely correlated with severity of disease and outcome in the post-arrest period and were shown to be a predictive marker of ICU mortality

## Abbreviations

AUC: area under the curve
CAD: coronary artery disease
CFDA: carboxyfluorescein-diacetate
CPR: cardiopulmonary resuscitation
ERK: extracellular signal-regulated kinases
FBS: fetal bovine serum
FCS: fetal calf serum
GOS: Glasgow outcome scale
GPx: glutathione peroxidase
HUVEC: human umbilical vein endothelial cells
ICAM-1: intracellular adhesion molecule-1
MPs: microparticles
PBS: phosphate-buffered saline
PCAS: post-cardiac arrest syndrome
PEA: pulseless electrical activity
ROC: receiver operating characteristic
ROS: reactive oxygen species
ROSC: return of spontaneous circulation
SEM: standard error of the mean
SIRS: systemic inflammatory response syndrome
SOFA: sequential organ failure assessment
TNF-α: tumor necrosis factor- α
VCAM-1: vascular cell adhesion molecule-1
VF: ventricular fibrillation
VT: ventricular tachycardia

## References

[1] Nichol G, Thomas E, Callaway CW, Hedges J, Powell JL, Aufderheide TP (2008). Resuscitation outcomes consortium investigators regional variation in out-of-hospital cardiac arrest incidence and outcome. JAMA.

[2] Sandroni C, Nolan J, Cavallaro F, Antonelli M (2007). In-hospital cardiac arrest: incidence, prognosis and possible measures to improve survival. Intensive Care Med.

[3] Negovsky VA (1972). The second step in resuscitation: the treatment of the “post-resuscitation disease”. Resuscitation.

[4] Peberdy MA, Callaway CW, Neumar RW, Geocadin RG, Zimmerman JL, Donnino M (2010). Part 9: post-cardiac arrest care: 2010 American heart association guidelines for cardiopulmonary resuscitation and emergency cardiovascular care. Circulation.

[5] Adrie C, Adib-Conquy M, Laurent I, Monchi M, Vinsonneau C, Fitting C (2002). Successful cardiopulmonary resuscitation after cardiac arrest as a “sepsis-like” syndrome. Circulation.

[6] Geppert A, Zorn G, Karth GD, Haumer M, Gwechenberger M, Koller-Strametz J (2000). Soluble selectins and the systemic inflammatory response syndrome after successful cardiopulmonary resuscitation. Crit Care Med.

[7] Gando S, Nanzaki S, Morimoto Y, Kobayashi S, Kemmotsu O (2000). Out-of-hospital cardiac arrest increases soluble vascular endothelial adhesion molecules and neutrophil elastase associated with endothelial injury. Intensive Care Med.

[8] Fink K, Schwarz M, Feldbrügge L, Sunkomat J, Schwab T, Bourgeois N (2010). Severe endothelial injury and subsequent repair in patients after successful cardiopulmonary resuscitation. Crit Care.

[9] Grundmann S, Fink K, Rabadzhieva L, Bourgeois N, Schwab T, Moser M (2012). Perturbation of the endothelial glycocalyx in post cardiac arrest syndrome. Resuscitation.

[10] Böttiger BW, Motsch J, Böhrer H, Böker T, Aulmann M, Nawroth PP (1995). Activation of blood coagulation after cardiac arrest is not balanced adequately by activation of endogenous fibrinolysis. Circulation.

[11] Combes V, Simon AC, Grau GE, Arnoux D, Camoin L, Sabatier F (1999). In vitro generation of endothelial microparticles and possible prothrombotic activity in patients with lupus anticoagulant. J Clin Invest.

[12] Ahn YS (2005). Cell-derived microparticles: “Miniature envoys with many faces”. J Thromb Haemost.

[13] Chironi GN, Boulanger CM, Simon A, Dignat-George F, Freyssinet JM, Tedgui A (2009). Endothelial microparticles in diseases. Cell Tissue Res.

[14] Satta N, Toti F, Feugeas O, Bohbot A, Dachary-Prigent J, Eschwège V (1994). Monocyte vesiculation is a possible mechanism for dissemination of membrane-associated procoagulant activities and adhesion molecules after stimulation by lipopolysaccharide. J Immunol.

[15] Diehl P, Fricke A, Sander L, Stamm J, Bassler N, Htun N (2012). Microparticles: major transport vehicles for distinct microRNAs in circulation. Cardiovasc Res.

[16] Diamant M, Tushuizen ME, Sturk A, Nieuwland R (2004). Cellular microparticles: new players in the field of vascular disease?. Eur J Clin Invest.

[17] Barry OP, Praticò D, Lawson JA, Fitzgerald GA (1997). Transcellular activation of platelets and endothelial cells by bioactive lipids in platelet microparticles. J Clin Invest.

[18] Sabatier F, Roux V, Anfosso F, Camoin L, Sampol J, Dignat-George F (2002). Interaction of endothelial microparticles with monocytic cells in vitro induces tissue factor-dependent procoagulant activity. Blood.

[19] Martin S, Tesse A, Hugel B, Martinez MC, Morel O, Freyssinet JM (2004). Shed membrane particles from T lymphocytes impair endothelial function and regulate endothelial protein expression. Circulation.

[20] Brodsky SV, Zhang F, Nasjietti A, Gollgorsky MS (2004). Endothelium-derived microparticles impair endothelial function in vitro. Am J Physiol Heart Circ Physiol.

[21] Fink K, Feldbrügge L, Schwarz M, Bourgeois N, Helbing T, Bode C (2011). Circulating annexin V positive microparticles in patients after successful cardiopulmonary resuscitation. Crit Care.

[22] Zhang F, Yu W, Hargrove JL, Greenspan P, Dean RG, Taylor EW (2002). Inhibition of TNF-alpha induced ICAM-1, VCAM-1 and E-selectin expression by selenium. Atherosclerosis.

[23] Li YB, Han JY, Jiang W, Wang J (2011). Selenium inhibits high glucose-induced cyclooxygenase-2 and P-selectin expression in vascular endothelial cells. Mol Biol Rep.

[24] Maddox JF, Aherne KM, Reddy CC, Sordillo LM (1999). Increased neutrophil adherence and adhesion molecule mRNA expression in endothelial cells during selenium deficiency. J Leukoc Biol.

[25] Özbal S, Erbil G, Koçdor H, Tuğyan K, Pekçetin C, Ozoğul C (2008). The effects of selenium against cerebral ischemia-reperfusion injury in rats. Neurosci Lett.

[26] Ostadalova I, Vobecky M, Chvojkova Z, Mikova D, Hampl V, Wilhelm J (2007). Selenium protects the immature rat heart against ischemia/reperfusion injury. Mol Cell Biochem.

[27] Guo F, Monsefi N, Moritz A, Beiras-Fernandez A (2012). Selenium and cardiovascular surgery: an overview. Curr Drug Saf.

[28] Hawker FH, Stewart PM, Snitch PJ (1990). Effects of acute illness on selenium homeostasis. Crit Care Med.

[29] Forceville X, Vitoux D, Gauzit R, Combes A, Lahilaire P, Chappuis P (1998). Selenium, systemic immune response syndrome, sepsis, and outcome in critically ill patients. Crit Care Med.

[30] Angstwurm MWA, Engelmann L, Zimmermann T, Lehmann C, Spes CH, Abel P (2007). Selenium in Intensive Care (SIC): results of a prospective randomized, placebo-controlled, multiple-center study in patients with severe systemic inflammatory response syndrome, sepsis, and septic shock. Crit Care Med.

[31] Manzanares W, Dhaliwal R, Jiang X, Murch L, Heyland DK (2012). Antioxidant micronutrients in the critically ill: a systematic review and meta-analysis. Crit Care.

[32] Boulanger CM, Scoazec A, Ebrahimian T, Henry P, Mathieu E, Tedgui A (2001). Circulating microparticles from patients with myocardial infarction cause endothelial dysfunction. Circulation.

[33] Lubos E, Sinning CR, Schnabel RB, Wild PS, Zeller T, Rupprecht HJ (2010). Serum selenium and prognosis in cardiovascular disease: results from the AtheroGene study. Atherosclerosis.

[34] Flores-Mateo G, Novas-Acien A, Pastor-Barriuso R, Guallar E (2006). Selenium and coronary heart disease: a meta-analysis. Am J Clin Nutr.

[35] Goon PKY, Lip GYH, Boos CJ, Stonelake PS, Blann AD (2006). Circulating endothelial cells, endothelial progenitor cells, and endothelial microparticles in cancer. Neoplasia.

[36] Sakr Y, Reinhart K, Bloos F, Marx G, Russwurm S, Bauer M (2007). Time course and relationship between plasma selenium concentrations, systemic inflammatory response, sepsis, and multiorgan failure. Br J Anaesth.

[37] Vincent JL, Moreno R, Takala J, Willatts S, De Mendonça A, Bruining H (1996). The SOFA (Sepsis-related Organ Failure Assessment) score to describe organ dysfunction/failure. On behalf of the Working Group on Sepsis-Related Problems of the European Society of Intensive Care Medicine. Intensive Care Med.

[38] Adrie C, Laurent I, Monchi M, Cariou A, Dhainaou JF, Spaulding C (2004). Postresuscitation disease after cardiac arrest: a sepsis-like syndrome?. Curr Opin Crit Care.

[39] Gambim MH (2007). do Carmo Ade O, Marti L, Veríssimo-Filho S, Rossetti Lopes L, Janiszewski M. Platelet-derived exosomes induce endothelial cell apoptosis through peroxynitrite generation: experimental evidence for a novel mechanism of septic vascular dysfunction. Crit Care.

[40] Barry OP, Praticò D, Savani RC, Fitzgerald GA (1998). Modulation of monocyte-endothelial cell interactions by platelet microparticles. J Clin Invest.

[41] Empana JP, Boulanger CM, Tafflet M, Renard JM, Leroyer AS, Varenne O, et al. Microparticles and sudden cardiac death due to coronary occlusion. The TIDE (Thrombus and Inflammation in sudden DEath) study. Eur Heart J Acute Cardiovasc Care. 2014 Jun 9 2014 Jun 9. pii: 2048872614538404 [Epub ahead of print]. 10.1177/2048872614538404 24912925

[42] Wang JG, Williams JC, Davis BK, Jacobson K, Doerschuk CM, Ting JPY (2011). Monocytic microparticles activate endothelial cells in an IL-1b-dependent manner. Blood.

[43] Rautou PE, Leroyer AS, Ramkhelawon B, Devue C, Duflaut D, Vion AC (2011). Microparticles from human atherosclerotic plaques promote endothelial ICAM-1- dependent monocyte adhesion and transendothelial migration. Circ Res.

[44] Conley SM, McKay BS, Gandolfi AJ, Stamer WD (2006). Alterations in human trabecular meshwork cell homeostasis by selenium. Exp Eye Res.

[45] Berger MM, Cavadini C, Chiolero R, Dirren H (1996). Copper, selenium, and zinc status and balances after major trauma. J Trauma.

[46] Stoppe C, Schälte G, Rossaint R, Coburn M, Graf B, Spillner J (2011). The intraoperative decrease of selenium is associated with the postoperative development of multiorgan dysfunction in cardiac surgical patients. Crit Care Med.

[47] Maddipati KR, Gasparski C, Marnett LJ (1987). Characterization of the hydroperoxide-reducing activity of human plasma. Arch Biochem Biophys.

[48] Holmgren A (1989). Thioredoxin and glutaredoxin systems. J Biol Chem.

[49] Fernando MR, Nanri H, Yoshitake S, Nagata-Kuno K, Minakami S (1992). Thioredoxin regenerates proteins inactivated by oxidative stress in endothelial cells. Eur J Biochem.

[50] Angstwurm MW, Schottdorf J, Schopohl J, Gaertner R (1999). Selenium replacement in patients with severe systemic inflammatory response syndrome improves clinical outcome. Crit Care Med.

[51] Nève J (1995). Human selenium supplementation as assessed by changes in blood selenium concentration and glutathione peroxidase activity. J Trace Elem Med Biol.

[52] Combs GF, Watts JC, Jackson MI, Johnson LK, Zeng H, Scheett AJ (2011). Determinants of selenium status in healthy adults. Nutr J.

[53] Landucci F, Mancinelli P, De Gaudio AR, Virgili G (2014). Selenium supplementation in critically ill patients: a systematic review and meta-analysis. J Crit Care.

[54] Alhazzani W, Jacobi J, Sindi A, Hartog C, Reinhart K, Kokkoris S (2013). The effect of selenium therapy on mortality in patients with sepsis syndrome: a systematic review and meta-analysis of randomized controlled trials. Crit Care Med.

[55] Huang TS, Shyu YC, Chen HY, Lin LM, Lo CY, Yuan SS (2013). Effect of parenteral selenium supplementation in critically Ill patients: a systematic review and meta-analysis. PLoS One.

[56] Stoppe C, Spillner J, Rossaint R, Coburn M, Schälte G, Wildenhues A (2013). Selenium blood concentrations in patients undergoing elective cardiac surgery and receiving perioperative sodium selenite. Nutrition.

